# Tablet computers for implementing NICE antenatal mental health guidelines: protocol of a feasibility study

**DOI:** 10.1136/bmjopen-2015-009930

**Published:** 2016-01-22

**Authors:** José S Marcano-Belisario, Ajay K Gupta, John O'Donoghue, Cecily Morrison, Josip Car

**Affiliations:** 1 Global eHealth Unit, Department of Primary Care and Public Health, Imperial College London, London, UK; 2 National Heart & Lung Institute, Imperial College London, London, UK

**Keywords:** MENTAL HEALTH, PUBLIC HEALTH

## Abstract

**Introduction:**

Depression is one of the most common mental health disorders that may affect women during pregnancy. The prompt identification of this disorder, and the provision of treatment, may help to reduce the likelihood of post-partum depression, prevent severe forms of the disease, and reduce its intergenerational impact. Despite women's repeated encounters with health services throughout their antenatal care, depression often goes undiagnosed. This is one area where mobile health could prove useful. We will assess the feasibility of using tablets to incorporate depression screening into antenatal pathways. We will also assess if survey layout could affect the quality of the data collected through these devices.

**Methods and analysis:**

We will test the feasibility of using iPad Airs for the administration of the Whooley questions and the Edinburgh Postnatal Depression Scale (EPDS) to pregnant women attending antenatal clinics in England. We will assess the impact of survey layout on the quality of the responses given to these screening scales using a parallel, randomised controlled study design. We will calculate the positive predictive value, the negative predictive value and the false omission rate of the Whooley questions in comparison with the EPDS. We will calculate differences in data equivalence, time needed to complete the surveys, break-off rates, data completeness and requests for help between the 2 experimental groups: using all questions in one screen and navigation by vertical scrolling, or a single question per screen and navigation by multiple pages.

**Ethics and dissemination:**

This study has been approved by the National Research Ethics Service Committee South East Coast—Surrey. Our findings will be disseminated through academic peer-reviewed publications, conferences and discussion with peers.

**Strengths and limitations of this study**

- This study addresses an important area of unmet clinical need, which has a considerable impact on healthcare systems and society in general.
- This study will attempt to identify implementation issues (arising from the introduction of an mHealth intervention into a clinical process) that could impair the ability of already busy healthcare professionals to perform their clinical duties. This is in contrast to some existing studies, where mHealth interventions are deployed in highly controlled environments that do not capture the realities of clinical settings.
- This study will explore the effect that survey presentation could have on the quality of the data collected (when using a validated scale). This is in contrast to other studies in the medical literature (that assess mHealth interventions), which have not explored how these additional factors could affect data quality.

- This study will explore mHealth as a delivery mode for a depression screening validated scale. However, it will not evaluate the potential of mHealth as a delivery mode for evidence-based interventions.
- Our findings might only be generalisable to devices with similar technical specifications that are being used in similar clinical settings.

## Introduction

Maternal mental health is a key public health priority due to the impact it can have on a woman's well-being and on her child's emotional, behavioural, cognitive and social development.^1–5^ Perinatal depression is one of the most common psychiatric disorders that can affect up to 15% of women during pregnancy or within 1 year of giving birth. The direct and indirect financial costs of perinatal depression to the health system, together with its direct impact on the mother and her child, account for the majority of the overall costs to society of perinatal mental disorders.^5^ In the UK, these costs have been estimated at £8.1 billion for each 1-year cohort of birth (with the average costs of one case of perinatal depression estimated at £74 k), 72% of which could be attributed to the adverse effects experienced by the child.^2^ For this reason, the early identification of women who might be at risk of developing or have perinatal depression should be considered a priority.^6^

Women appear to be at an increased risk of depression 3–6 months after childbirth,^1^ ^4^ although a recent longitudinal study found that symptoms of depression may be more prevalent at 4 years post-partum.^7^ Several factors are known to increase the likelihood of developing post-partum depression.^1^ ^6^ ^8^ Depression during pregnancy, however, has been recognised as one of the most important risk factors and one that is susceptible to early intervention. The point prevalence of depression during the first, second and third trimesters of pregnancy has been estimated at 7.4%, 12.8% and 12%, respectively.^9^ The timely identification of depression during these stages, and the provision of appropriate treatment, can reduce the likelihood of developing post-partum depression, prevent more severe forms of this disorder, and improve a woman's general health status.^4^ ^5^ Treating depression during pregnancy may also help reduce the intergenerational impact of this condition.

Nonetheless, depression during pregnancy often goes undiagnosed and its routine screening is still debated.^10^ Validated screening tools, such as the Edinburgh Postnatal Depression Scale (EPDS), can facilitate the early identification of depressive symptoms in pregnant women.^11^ However, cost-effectiveness is often cited as one of the key reasons against the routine screening for this condition.^12^ This is an area where smartphones and tablet computers could demonstrate their value in a field of study that is known as mobile health (mHealth). Thanks to their advanced computational and networking capabilities, and overall popularity (in the first quarter of 2015, it was reported that approximately 66% of adults in the UK owned a smartphone),^13^ these devices could facilitate depression screening and monitoring of symptoms^14^ while reducing costs and administrative burden.

Researchers from the School of Social Work at the University of Illinois and staff members at Champaign-Urbanara Public Health District will be testing the use of tablet computers to implement depression screening in maternal clinics.^15^ They will be doing so during antenatal consultations. Mobile devices could be used to screen for depression before a consultation, thus releasing some of the consultation time. For this reason, we need to establish the feasibility of using these devices to do so.

One aspect of delivering depression screening through mobile devices is the impact that this technology may have on data quality. This is to ensure that the data accurately reflect underlying clinical changes. Our recent Cochrane review^16^ suggests that collecting patient self-reported data through apps is equivalent to collecting data through alternative delivery modes (ie, paper, short messaging service, laptops, plastic rulers and personal digital assistants). This review also highlighted that equivalence, and other data quality dimensions, could be affected by factors other than the delivery mode.^16^ One such factor is survey presentation. Unlike the survey methodology literature, however,^17^ ^18^ the effect of this factor on validated clinical tools has not been systematically explored in the medical literature.

In this study, we will assess the feasibility of implementing the recommendations from The National Institute for Health and Care Excellence (NICE)^19^ for recognising depression during pregnancy. We will conduct our study in the waiting area of general practices, community midwiferies and hospitals using iPad Air tablets. We will evaluate the statistical properties of one of the validated instruments recommended by this guideline. We will also assess if survey layout (as a component of survey presentation) affects the quality of the responses collected through the screening instruments under evaluation.

## Methods and analysis

### Study design

We will assess the feasibility of using iPad Air tablets to administer a brief survey asking for personal information, the Whooley questions and the EPDS (see online supplementary appendix 1) to pregnant women attending antenatal clinics across a number of primary and secondary care National Health Service (NHS) centres in England. iPad Air tablets have a 9.7-inch (diagonal) retina display, with a 2048-by-1536 resolution at 264 pixels per inch. Study data will be collected and managed using Snap survey tools.^20^ Snap Mobile app V.4.0.30 (or later, if a new version becomes available) will be running on iOS V.9.0.2 (or later, if a new version becomes available).

In order to evaluate the effect of survey layout on data quality, we will use a parallel, randomised controlled study design. Pregnant women consenting to take part in this study will be randomly assigned to complete (1) an app version of the Whooley questions and the EPDS in which all the questions are presented on a single screen (app screening—scrolling layout), causing participants to scroll vertically on the device; or (2) an app version of the Whooley questions and the EPDS in which only one question per page is presented at any given time (app screening—paging layout), causing participants to navigate through multiple pages.

### Pilot phase

We piloted both versions of the surveys used in this study (paging layout and scrolling layout) with four staff members at the Department of Primary Care and Public Health at Imperial College London. This phase allowed us to identify (and rectify) the following issues:

- Font size;
- Consistency of response formats (in particular, observed differences between yes–no questions and multiple choice questions with a single answer);
- Framing of the survey results.

### Sample selection and recruitment

We will select our sample of participants from pregnant women attending antenatal clinics in general practices, midwifery services and hospitals ,which are part of the NHS in England. Each potential participant will be assessed by clinical staff against our inclusion and exclusion criteria (table 1).

**Table 1:** Participant inclusion and exclusion criteria

Inclusion criteria	Exclusion criteria
Women who are 18 years old or older	Current diagnosis of mood (affective) disorders as specified in the ICD-10, Classification of Mental Health and Behavioural Disorders^21^
Pregnant and of any gestational age	Currently receiving treatment for mood (affective) disorders
Any parity	Recent personal history of mood (affective) disorders within the past 12 months
Attending antenatal clinics in participating NHS sites	Not comfortable reading and writing in English

ICD-10, 10th revision of the International Statistical Classification of Disease and Related Health Problems; NHS, National Health Service.

On the day in the antenatal clinic, potential participants will be first approached in the waiting room of the participating centres by a member of the clinical care team. If a potential participant expresses her interest in this study, she will be introduced to one of our researchers who will explain the details of the study to her. Potential participants will be provided with a patient information sheet. The researcher will emphasise that the potential participant can ask any questions regarding the study. If a potential participant agrees to take part in this study, she will be asked to complete a consent form, followed by the study procedures (ie, personal demographic survey, Whooley questions, and Edinburgh Postnatal Depression Scale). Otherwise, if she is unsure about participation, she will have at least 24 h to decide. In this case, she would only take part in the study if she comes into contact with the research team during a future antenatal clinic.

Refusal to take part in this study will not have an impact on a woman's legal rights, medical care or on the relationship with her care providers. We will obtain written, informed consent from those women who, after receiving all the relevant study information and asking study-related questions, still wish to take part in this study.

### Interventions to be measured

#### Surveys

##### Non-validated, personal demographic survey

We will administer an 11-question survey to collect information about the woman's age group, ethnic background, marital status, employment status, level of education, smartphone or tablet computer ownership, parity, and previous personal history of depression.

##### Whooley questions

The Whooley questions were developed as a case-finding instrument for depression in primary care.^22^ This two-question instrument assesses depressed mood and anhedonia that have been present during the past month. Respondents are required to answer *yes* or *no* to each question.

##### Edinburgh Postnatal Depression Scale

The EPDS is a 10-item self-administered survey that was developed to screen for perinatal depression in the community.^23^ This instrument assesses feelings of guilt, sleep disturbance, reduced energy levels, anhedonia and suicidal ideation that have been present during the past 7 days. Each question is scored on a four-point scale ranging from 0 to 3 points. An overall score is calculated by adding the scores from each individual question. Overall scores between 10 and 12 points suggest increased risk for depression; scores of 13 points or more suggest that the diagnostic criteria for major depression disorder have probably been met.^24^ In addition, special attention should be paid to item 10, as it deals with suicidal thoughts. On the basis of these scores, a clinician would be prompted to refer a woman to a mental health professional.

The EPDS is a valid and reliable tool for identifying women who are at risk of depression, both during pregnancy and post-partum. This instrument is also sensitive to changes in the severity of depression over time.^23^ The EPDS can be reproduced without further permission provided that the original source of the scale is cited in each reproduced copy.

#### Screening for depression and effect of survey layout

Pregnant women taking part in our study will receive verbal instructions on how to complete the surveys. Participants will also be informed that we will communicate their answers to the Whooley questions and their scores on the EPDS to their clinicians, and that these results will be available for discussion during their antenatal appointment. Participants’ results on the Whooley questions and on the EPDS will be communicated to their general practitioners.

The researcher will also explain to participants that they can ask for help at any point while completing the surveys, and that all questions should be directed to a member of the research team (not to the clinical care team). We will offer help with the use of the device. However, we will not offer help reading questions out loud to participants (as the Whooley questions and the EPDS are intended to be used as self-administered instruments).

Participants will be asked to complete all the surveys while waiting for their appointment in the waiting room of participating NHS sites. They will be randomised to one of two experimental manipulations of the survey layout.

##### Scrolling layout

**Figure 1:**
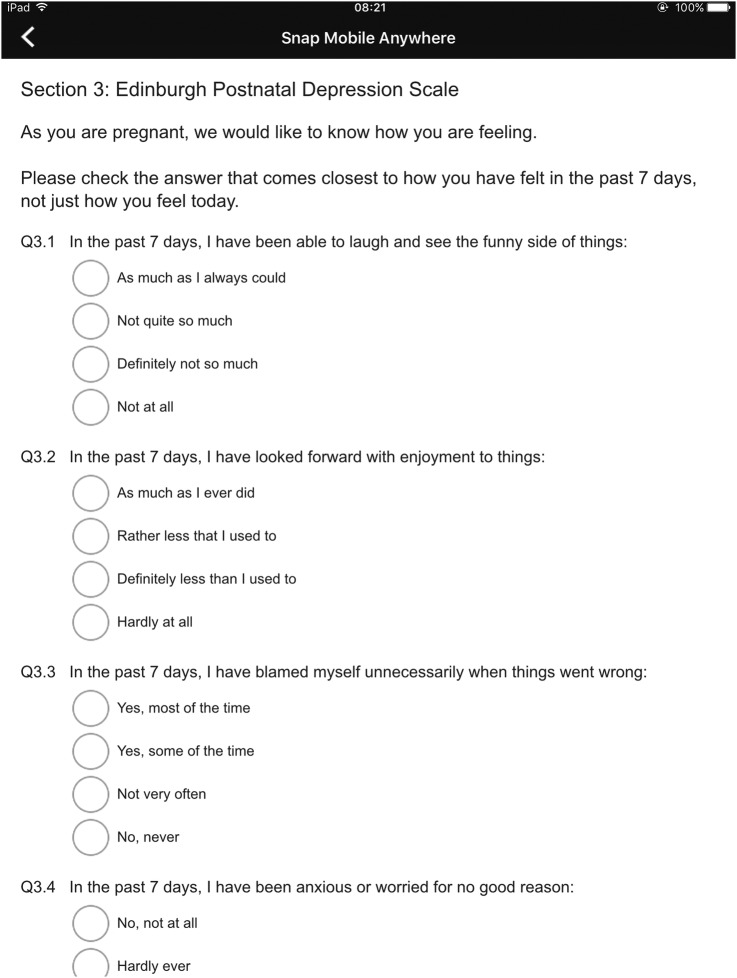
Scrolling layout (screenshot).

In this experimental manipulation, all the surveys will be presented on a single screen (figure 1). Therefore, participants will need to scroll vertically in order to answer all the questions. They will be able to scroll up and down as they wish and to modify the answers given to previous questions as long as they do it before submitting their answers. Once a participant has submitted her answers, a message acknowledging their participation will be displayed on the screen of the device and they will no longer be able to modify their answers. Once all the surveys have been completed, participants will be asked to return the iPad Air to a member of the research team. Validation procedures built into Snap surveys will ensure that participants are not able to submit unanswered questions.

##### Paging layout

**Figure 2:**
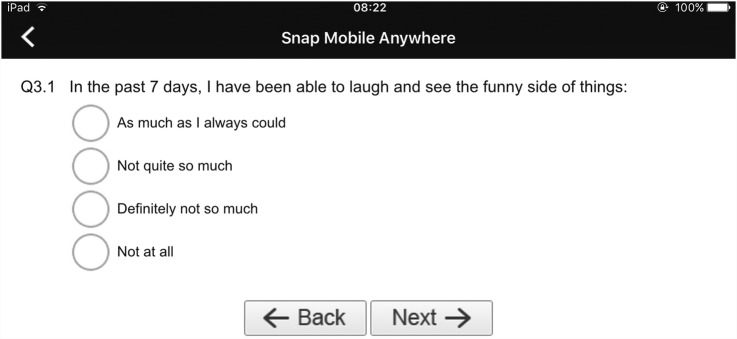
Paging layout (screenshot).

In this experimental manipulation, participants will only see one question on the screen at any given time (figure 2). They will need to select and submit their answer to the current question before being able to move on to the next question. Participants will not be allowed to revisit already answered questions. Once a participant has submitted all her answers, a message acknowledging her participation will be displayed on the screen of the device. Once all the surveys have been completed, participants will be asked to return the iPad Air to a member of the research team. Validation procedures built into Snap surveys will ensure that participants are not able to submit unanswered questions.

### Randomisation

We will utilise a block randomisation procedure to allocate participants to one of the two experimental arms. Random numbers will be generated using Stata V.13.0 (Stata. Stata Statistical Software[Program]. Version 13, 2013). Each consecutive number that is generated will be printed and put inside an opaque envelope, which will then be sealed. Envelopes will be numbered in an ascending sequence. Once a participant has provided informed consent, the researcher will take the relevant envelope, open it and use the number contained in it to allocate the participant to one of the two experimental groups. Researchers conducting participant recruitment will not be involved in this randomisation procedure in order to avoid recruitment bias.

### Outcomes

#### Positive predictive value of the Whooley questions

We will calculate the number of pregnant women who answered *yes* to any of the Whooley questions AND who scored 10 points or higher on the EPDS, as a proportion of the total number of pregnant women who answered *yes* to any of the Whooley questions regardless of their EPDS scores.

#### Negative predictive value of the Whooley questions

We will calculate the number of pregnant women who answered *no* to both Whooley questions AND who scored nine points or less on the EPDS, as a proportion of the total number of pregnant women who answered *no* to both Whooley questions regardless of their EPDS scores.

#### False omission rate of the Whooley questions

We will calculate the number of pregnant women who answered *no* to both Whooley questions AND who scored 10 points or higher on the EPDS, as a proportion of the total number of pregnant women who answered *no* to both Whooley questions regardless of their EPDS scores.

#### Mean overall scores on the EPDS

For each experimental group, we will sum the individual scores on the EPDS and divide the total by the number of participants in the group. We will also calculate the mean overall scores on the EPDS for our total sample.

#### Break-off rates

We will calculate the proportion of participants who interrupt the survey before reaching the end. In such eventualities, we will document the reason for the break-off.

#### Time needed to complete surveys

We will measure the time in seconds between a participant starting to read the basic instructions on how to complete the survey and the participants receiving the message acknowledging their participation. We will also account for the time that participants spend experiencing problems, distractions or making requests for help or clarification.

#### Proportion of complete EPDS records

We will define a complete record as one in which no question was left unanswered. Leaving unanswered questions will not be possible due to the implementation of data validation procedures. Therefore, data completeness will be a by-product of break-off rates.

#### Proportion of participants requesting help

We will document the requests for help made by our participant. We will categorise participants into three groups: (1) one request for help; (2) between two and four requests for help; and (3) five or more requests for help. We will compute the proportion of participants falling within each category.

#### Proportion of each type of request for help

We will document the reasons for each request for help. This information will be captured through a post study procedures survey that researchers will have to complete. We will conduct a content analysis of these requests in order to categorise them according to their nature.

### Sample size calculations

We powered our study according to the positive predictive value (PPV), negative predictive value (NPV) and false omission rate (FOR) of the Whooley questions. In order to calculate these outcomes, we would need at least 30 events (defined as pregnant women scoring 10 points or more on the EPDS). Assuming a prevalence of depression in pregnant women of 12%, we would need to recruit 250 participants in our study. We decided to recruit a total of 300 participants to account for any eventualities, such as dropouts.

### Data analysis plan

#### Descriptive statistics

We will report the number of participants approached who did not meet our inclusion criteria, as well as the reason for exclusion. We will also report the number of participants who refused to take part in our study as a proportion of the total number of participants who were initially approached.

We will report the following information for each experimental group:

- Demographic characteristics (as captured by our non-validated survey);
- Proportion of participants answering *Yes* to any of the Whooley questions;
- Proportion of participants scoring at each interval of the EPDS: between 0 and 9 points; between 10 and 12 points; and 13 points or above;
- Proportion of participants scoring 2 or 3 points on question 10 of the EPDS;
- Mean time taken to complete the surveys;
- Proportion of participants making requests for help;
- Proportion of participants making each type of request;
- Mean time spent dealing with requests for help; and
- Break-off rates and reasons for break-off.

#### Inferential statistics

##### PPV, NPV and FOR

We will calculate the PPV, NPV and FOR of the Whooley questions for the total sample of participants, and will report these values as percentages. We will also test for significant differences between the two experimental groups in these outcomes using a t test.

##### Data equivalence

We will determine data equivalence between the experimental groups (scrolling and paging) by testing for statistically significant differences in the mean overall scores on the EPDS. For this, we will conduct a t test. In addition, we will compare the proportion of women scoring (1) between 10 and 12 points, and (2) 13 points or more on the EPDS between the two experimental groups using a t test.

##### Time needed to complete surveys

We will compare the mean time needed by participants to complete the survey questionnaire between the experimental groups using a t test.

##### Data completeness

We will compare the proportion of complete records between the experimental groups using a t test.

##### Proportion of requests for help

We will compare the proportion of participants making requests for help between the experimental groups. For this, we will conduct a t test for each category in this end point: (1) one request; (2) between two and four requests; and (3) five or more requests.

### Timeline

We expect recruitment of participants to take place between October 2015 and the end of February 2016.

## Conclusion

We believe that this study addresses an important area of unmet clinical need which has a direct and indirect impact on health systems and society. It will also contribute to building the evidence base for mHealth, particularly when applied to mental health. Unlike other studies in the medical literature, this study will evaluate how survey layout could affect the quality of the responses collected using a validated scale. Evaluating how different factors could affect data quality becomes important when data are used to inform clinical and therapeutic decisions. In addition, this study will also evaluate some of the implementation issues that might arise when deploying an mHealth intervention, and that could affect its successful adoption.

The technical specifications of a device, such as screen size, could influence user interaction and thus the quality of responses. In addition, usage patterns (in terms of the type of interactions, duration and frequency of an interaction, and the type of activities that occur during an interaction) vary depending on the type of device and the setting in which it is being used. For these reasons, our findings might only be generalisable to iPad Air tablets (or devices with similar technical specifications) that are being used in clinical settings. Therefore, in a future study, we will explore issues associated with the at-home collection of mood data during pregnancy, using participants’ own devices.

The findings from this feasibility study will inform a larger trial evaluating the integration of this intervention into routine antenatal pathways, and the potential impact on clinical outcomes.

## Ethics and dissemination

This study has been reviewed and approved by the National Research Ethics Service (NRES) Committee South East Coast—Surrey on 9 July 2015 under the Research Ethics Committee (REC) reference 15/LO/0977.

This study is being sponsored by Imperial College London under the reference number 15IC2687 and has been included in the UK Clinical Research Network (CRN) Study Portfolio under the UKCRNID number 19280.

Lastly, this study protocol has been registered in ClinicalTrials.gov under the identifier NCT02516982 (as required by the REC).

The findings of this study will be disseminated through academic peer-reviewed publications, poster presentations and abstracts at academic and professional conferences, discussion with peers, social media and the Global eHealth Unit website. The findings of this study will also inform JSM-B's PhD thesis.

## References

[1] Abdollahi F, Zarghami M, Azhar MZ Predictors and incidence of post-partum depression: a longitudinal cohort study. J Obstet Gynaecol Res 2014;40:2191–200. 10.1111/jog.12471 25132641

[2] Bauer A, Parsonage M, Knapp M The costs of perinatal mental health problems. London: Centre for Mental Health and London School of Economics, 2014.

[3] Satyanarayana VA, Lukose A, Srinivasan K Maternal mental health in pregnancy and child behavior. Indian J Psychiatry 2011;53:351–61. 10.4103/0019-5545.91911 22303046 PMC3267349

[4] Yazici E, Kirkan TS, Aslan PA Untreated depression in the first trimester of pregnancy leads to postpartum depression: high rates from a natural follow-up study. Neuropsychiatr Dis Treat 2015;11:405–11. 10.2147/NDT.S77194 25737636 PMC4344179

[5] Thomas N, Komiti A, Judd F Pilot early intervention antenatal group program for pregnant women with anxiety and depression. Arch Womens Ment Health 2014;17:503–9. 10.1007/s00737-014-0447-2 25074561

[6] Parker GB, Hegarty B, Paterson A Predictors of post-natal depression are shaped distinctly by the measure of ‘depression’. J Affect Disord 2015;173:239–44. 10.1016/j.jad.2014.10.066 25462423

[7] Woolhouse H, Gartland D, Mensah F Maternal depression from early pregnancy to 4 years postpartum in a prospective pregnancy cohort study: implications for primary health care. BJOG 2015;122:312–21. 10.1111/1471-0528.12837 24844913

[8] Mohamad Yusuff AS, Tang L, Binns CW Prevalence and risk factors for postnatal depression in Sabah, Malaysia: a cohort study. Women Birth 2015;28:25–9. 10.1016/j.wombi.2014.11.002 25466643

[9] Bennett HA, Einarson A, Taddio A Prevalence of depression during pregnancy: systematic review. Obstet Gynecol 2004;103:698–709. 10.1097/01.AOG.0000116689.75396.5f 15051562

[10] Tsai AC, Tomlinson M, Dewing S Antenatal depression case finding by community health workers in South Africa: feasibility of a mobile phone application. Arch Womens Ment Health 2014;17:423–31. 10.1007/s00737-014-0426-7 24682529 PMC4167933

[11] Hewitt C, Gilbody S, Brealey S Methods to identify postnatal depression in primary care: an integrated evidence synthesis and value of information analysis. Health Technol Assess 2009;13:1–145, 147–230 10.3310/hta13360 19624978

[12] Paulden M, Palmer S, Hewitt C Screening for postnatal depression in primary care: cost effectiveness analysis. BMJ 2009;339:b5203 10.1136/bmj.b5203 20028779 PMC2797050

[13] Ofcom. Facts & Figures: Secondary Facts & Figures 2015. http://media.ofcom.org.uk/facts/ (accessed 18 Aug 2015).

[14] BinDhim NF, Shaman AM, Trevena L Depression screening via a smartphone app: cross-country user characteristics and feasibility. J Am Med Inform Assoc 2015;22:29–34. 10.1136/amiajnl-2014-002840 25326599 PMC4433364

[15] Pineros-Leano M, Tabb KM, Sears H Clinic staff attitudes towards the use of mHealth technology to conduct perinatal depression screenings: a qualitative study. Fam Pract 2015;32:211–15. 10.1093/fampra/cmu083 25535280 PMC7340322

[16] Marcano Belisario JS, Jamsek J, Huckvale K Comparison of self-administered survey questionnaire responses collected using mobile apps versus other methods. Cochrane Database Syst Rev 2015;7:MR000042 10.1002/14651858.MR000042.pub2 PMC8152947 26212714

[17] Mavletova A, Couper M Mobile web survey design: scrolling versus paging, SMS versus e-mail invitations. J Surv Stat Methodol 2014;2:498–518. 10.1093/jssam/smu015

[18] Wells T, Bailey JT, Link MW Comparison of smartphone and online computer survey administration. Soc Sci Comput Rev 2014;32:238–55. 10.1177/0894439313505829

[19] National Collaborating Centre for Mental Health. Antenatal and postnatal mental health: clinical management and service guidance—updated edition. Leicester and London: The British Psychological Society and The Royal College of Psychiatrists, 2014. 26180865

[20] Snap® surveys. http://www.snapsurveys.com/ (accessed 14 Oct 2015).

[21] The World Health Organization. The ICD-10 classification of mental and behavioural disorders: clinical descriptions and diagnostic guidelines. Geneva: World Health Organization, 1992.

[22] Whooley MA, Avins AL, Miranda J Case-finding instruments for depression—two questions are as good as many. J Gen Intern Med 1997;12:439–45. 10.1046/j.1525-1497.1997.00076.x 9229283 PMC1497134

[23] Cox JL, Holden JM, Sagovsky R Detection of postnatal depression—development of the 10-item Edinburgh Postnatal Depression Scale. Br J Psychiatry 1987;150:782–6. 10.1192/bjp.150.6.782 3651732

[24] Allbaugh LJ, Marcus SM, Ford EC Development of a screening and recruitment registry to facilitate perinatal depression research in obstetrics settings in the USA. Int J Gynaecol Obstet 2015;128:260–3. 25468049 10.1016/j.ijgo.2014.09.015

